# Reducing amount and frequency of meal as a major coping strategy for food insecurity

**DOI:** 10.1186/s13690-018-0303-3

**Published:** 2018-10-04

**Authors:** Adino Tesfahun Tsegaye, Amare Tariku, Abebaw Gebeyehu Worku, Solomon Mekonnen Abebe, Mezgebu Yitayal, Tadesse Awoke, Kassahun Alemu, Gashaw Andargie Biks

**Affiliations:** 1 Department of Epidemiology and Biostatistics, Institute of Public Health, College of Medicine and Health Sciences, University of Gondar, Gondar, Ethiopia; 2 Department of Human Nutrition, Institute of Public Health, College of Medicine and Health Sciences, University of Gondar, Gondar, Ethiopia; 3 Department of Health Service Management and Economics, Institute of Public Health, College of Medicine and Health Sciences, University of Gondar, Gondar, Ethiopia; 4 Department of Reproductive Health, Institute of Public Health, College of Medicine and Health Sciences, University of Gondar, Gondar, Ethiopia; 5 Dabat Research Centre Health and Demographic Surveillance System, Institute of Public Health, College of Medicine and Health Sciences, University of Gondar, Gondar, Ethiopia

**Keywords:** Food insecurity, Coping, Reducing amount and frequency of meal, Food aid, Borrowing, Ethiopia

## Abstract

**Background:**

Food insecurity is a global problem affecting many people worldwide, including approximately 220 million people in sub-Saharan Africa. Ethiopia is among the countries severely affected by hunger. However, evidence on how populations within Ethiopia cope with hunger and food insecurity is limited. This study aimed to identify household coping mechanisms in response to food insecurity at a Dabat Health and Demographic Surveillance System site.

**Methods:**

This study used data from a re-census collected between October 2014 and December 2014.15,159 household members in thirteen kebeles of the Dabat Health and Demographic surveillance system were included. The outcome variables of the study were food insecurity and coping strategies. Household Food Insecurity Access Scale (HFIAS) was used to assess food insecurity. If food insecurity was found, families were asked about coping mechanisms used. Binary logistic regression analysis was applied to identify socio-demographic determinants of reducing amount and frequency of meal as a coping mechanism in response to food insecurity.

**Result:**

Of the 15,159 households surveyed, 6671 (44.01%) reported the presence of a food insecurity in their household. Decreasing meal frequency and portions (3733 (55.96%)), borrowing money and food (2542 (38.11%)), and receiving food and money aid (1779 (26.67%)) were among the major coping strategies used by the households. Urban dwellers (AOR 2.07: 95% CI 1.74, 2.46), mid-altitude *(weyina-dega*) and high-land *(dega)* dwellers (AOR 2.46: 95% CI 2.08, 2.92 and AOR 1.22 95% CI 1.08, 1.38 respectively), and not married persons (AOR 1.60: 95% CI 1.07, 2.39) were more likely to consume less when faced with a food insecurity (using reducing amount and frequency of meal as a coping strategy).

**Conclusion:**

Households in the study area experienced a very high rate of food insecurity**.** Decreasing meal frequency and portions was the primary coping mechanism used by the households. Due to the severe insecurity of food in their household, many people chose to reduce the amount and frequency of their meal in order to prolong the small amount of food in their house. This finding indicates a high risk for undernourishment which can exacerbate the burden of malnutrition and related diseases in the region.

## Background

Food security is a global concern and describes a state when all people, at all times, have physical and economic access to sufficient, safe and nutritious food which can meet their dietary needs [1, 2]. Currently, food insecurity is a major challenge worldwide with17% of people in developing countries living in extreme poverty [3]. In 2015, the Food and Agricultural Organization (FAO) reported that hunger affects 795 million people worldwide and 780 million of those people live in the world’s developing regions. In sub-Saharan Africa, there were approximately 220 million hungry people in 2014–16 (23.2% of the population). East Africa, where Ethiopia is located, had the largest hunger burden in the region with an estimated 124 million undernourished people [4].

Many of the people most affected by hunger live in rural areas and rely on agriculture for much of their income [3]. Ensuring food security and appropriate nutrition within the poorest households is a special challenge since rural food production is limited by marginal soil fertility and a lack of resources to invest in buying the necessary agricultural inputs. In Ethiopia, more than 80% of the people live in rural areas where farming is the primary occupation. Agricultural productivity is dependent on rain and is highly vulnerable to climate change. Due to changes in weather patterns, the country has faced many droughts in recent years, which decreases crop productions and exacerbates food insecurity [2, 5].

The primary manifestation of food insecurity is food insecurity at the household level. As a result of food insecurity, these households face many social, economic, political and health consequences. The challenge of feeding family members has a variety of features and consequences [6] such as: migration and related health risks. Following food insecurity, people could have suicidal ideation [7], depression [8], undernutrition [9], obesity [10], diabetes mellitus [11], iron deficiency anemia [12], and deficiency of other nutrients [13]. In response to food insecurity, households use a range of mechanisms to cope.

Household coping mechanisms can vary due to season, geographic location, and other socio-economic factors [14]. A review of past literature reveals a variety of coping mechanisms used by the population during periods of food insecurity, including changing feeding habits by decreasing the amount and frequency of food intake [14–16], skipping a whole day without food [14, 15], borrowing food and money, [5, 6, 14–17], food aid [15], selling property [16], migration [5, 6], and harvesting fast-growing crops [15].

Farmers who rely on seasonal rains may also engage in other income generating activities to mitigate the risks and impacts of climate change on food security, such as owning a kiosk or small shop, selling local brews, providing traditional healing, creating artisan crafts, keeping livestock, being employed in some sectors, producing and selling natural resources (such as charcoal), and doing home gardening [18, 19]. Some people will eat wild plants during a time of food insecurity, which may have negative health impacts [5, 20]. The mechanism for coping with food insecurity may vary in association with marital status, household size, sex of household heads, [17] and other factors.

Despite the high level of food insecurity in Ethiopia [21, 22], there is limited information about coping mechanisms and the factors associated with certain coping methods used during a food insecurity particularly in the study area. Therefore, this study aims to identify and describe the coping mechanisms reported during periods of food insecurity at the Dabat Health and Demographic Surveillance System (DHDSS) site, with the intention of informing strategies to improve food security in Ethiopia. The findings of the study are expected to contribute towards filling the existing literature gap on understanding the food insecurity and coping strategies in Northwest Ethiopia. Since food security is one of the main national strategies, the findings in this study are expected to provide useful information for related policy formulation. This information is also would help to allocate resources and track the impact of interventions.

## Methods

### Study setting and design

The Dabat Health and Demographic Surveillance System (HDSS) site is located in Dabat District, which is a rural part of the Amhara regional state in northwest Ethiopia. Dabat HDSS is a full member of the International Network of Demographic Evaluation of Populations and Their Health (INDEPTH), a network of 44 HDSSs from the Global South. The Dabat District is located about 1000–3000 m above sea level. Dabat district was purposively selected as a surveillance site due to its unique three climatic conditions; *Dega* (high altitude and cold), *Woina dega* (mid-altitude and temperate) and *Kolla* (low altitude and hot). Researchers made this decision under the assumption that there would be significant differences in morbidity and mortality in the different climatic areas. After stratification of kebeles (smallest administrative units) by climatic zones, thirteen kebeles (nine rural and four urban) were selected.

Since the establishment of the Dabat HDSS site in November 1996, information on vital events has been collected every six months, with verbal autopsies (VAs) completed after reported deaths. The data used in this study was re-census data of 15,159 households in the DHDSS site that was collected between October 2014 and December 2014. All potential households in the selected kebeles were included in the data collection. The detailed data collection system, data quality control, the database, and the study setting of Dabat HDSS are described at the website of the University of Gondar [23].

### Study variables

The outcome variables of the study were food insecurity and coping strategies. Each household was asked about the existence of food insecurity to feed the family members and the respective coping mechanisms they used in response to any such insecurity in the four week period prior to the data collection time. To assess food insecurity: access, we used Household Food Insecurity Access Scale (HFIAS). The nine questions had three domains and they produce a total score between 0 and 27 with the higher score indicating greater food insecurity. Households were categorized into food secure and insecure, and food insecure households were further categorized as mildly, moderately or severely food insecure depending on the number of positive responses to questions related to severe conditions [24]. Food insecure households were further asked about the strategy they used to cope-up with the problem. Household Hunger Scale (HHS) was also calculated by using household hunger scale measurement guide [25]. Socio-demographic factors considered in the study included the following: sex of the household head, household wealth index, place of residence, household size, marital status of household head, religion, household head’s level of education, altitude/climatic zone, and household head’s occupation.

### Data collection

All households in nine rural kebeles and four urban kebeles from within Dabat District were contacted during the data collection period. A structured and pre-tested questionnaire was used to collect the data. The English version questionnaire was adapted and translated into Amharic, the national language. Respondents of the question were mainly females since they are culturally responsible to manage the household food. Trained and experienced HDSS site data collectors and supervisors carried out the data collection process. A total of 30 data collectors, 13 field supervisors, and 50 local guiders were recruited and involved in the data collection processes. All of the filed assistants (data collectors and supervisors) were permanent employees of the HDSS. The questionnaire was piloted outside the study area. Clarity of questions, applicability of tools, and procedures were evaluated by the pretest. To minimize recall bias, data collectors used different probing mechanisms as described in the guideline we used [24].

### Data management and analysis

Data were entered in the database using the household registration system software (HRS) version 2.1, and exported to STATA 14.0 for further analysis. Households were divided into socioeconomic quartiles based on their responses. The wealth status was assessed by household (HH) wealth index questions which were taken from the Ethiopian Demographic and Health Survey (EDHS) 2011 report [26]. It was divided into socioeconomic quantiles based on their scores. In order to capture wealth differences between urban and rural residences, the Principal Component Analysis (PCA) scores were generated for the two areas (urban and rural HH wealth indices) separately. In the principal component analysis, the power of the variables to explain wealth status was determined step by step using the communalities values. Those variables having communality value of greater than 0.5 were used to produce factor scores. Hence, an Eigen value of greater than one was considered. Finally, this factor scores were summed and ranked in to tertile as low, medium and better/high.

All individual descriptors collected referred to household heads, except questions regarding food insecurity, which included every member of each household (HH). Data from15,159 HHs located in the Dabat HDSS site were used. Descriptive measures were used to present household characteristics, to measure the magnitude of food insecurity, and to identify coping strategies in the study area. Binary logistic regression analysis was used to identify socio-demographic determinants of using reducing amount and frequency of meal as a coping mechanism. We had seven household related variables and since we believe that they have public health importance, we have incorporated all of them in the model. Hosmer–Lemeshow goodness-of-fit test was used to test the overall goodness-of-fit and the *P*-value was 0.1327 (non-significant and it indicates the model was fit). Adjusted odds ratio (AOR) with 95% confidence interval (CI) was used to report the strength of the association between reducing amount and frequency of meal and its explanatory variables.

### Ethical approval and consent to participate

The HDSS site received ethical clearance from Research Ethics Review Committee of University of Gondar, Ethiopian Public Health Association (EPHA), and US Center for Disease Control and Prevention (CDC). To capture occurrence of events to any family member, the head of family or an eligible adult among the family was interviewed. At the beginning of the surveillance system in 1996, written consents were obtained from each household in the surveillance site, and additional consent was obtained from new immigrants. For the consecutive data collection periods, we just obtain informed verbal consents. For this particular study, informed verbal consent was obtained from head of the family or eligible adult among the family. This consent procedure was outlined in the proposal, which was approved by the ethical review committees. To maintain confidentiality, data containing personal identifiers of subjects were not shared to third party.

## Results

### Socio-demographic characteristics of the study participants

A total of 15,159 households participated in this study. The mean age of the household head was 45 years (standard deviation (SD) ±16.3). 10,898 (71.89%) of the households were led by males and 9949 (65.63%) of the participants lived in rural areas. The average family size in the area was 4.2. Agriculture was the dominant source of income in the study area (68.2%) and the educational level of household heads is very low (Table 1).

**Table 1:** Socio demographic characteristics of the study, DHDSS, 2016

Variables	Frequency	Percent
Sex of the household head		
Female	4261	28.11
Male	10,898	71.89
Residence		
Rural	9949	65.63
Urban	5210	34.37
Household size		
1–4	8711	57.47
5–8	6056	39.94
9+	392	2.59
Wealth status		
Low income	4293	28.52
Middle income	5862	38.94
Better income	4898	32.54
Marital status of household head		
Married	10,048	66.29
Single	1202	7.93
Divorced	1435	9.47
Widowed	1901	12.54
Separated	573	3.78
Religion		
Orthodox	14,574	96.14
Islam	582	3.84
Others	3	0.02
Household Head’s level of education		
Unable to read and write	7428	49
Able to read and write	3395	22.4
Primary education	1623	10.71
Secondary education	1764	11.64
Tertiary education	949	6.26
Occupation		
Student	731	4.82
Farmer	10,339	68.2
All types of paid job	3345	22.07
Unemployed	320	2.11
^a^Others	424	2.8

^a^Others: Beggars, retired, who get different aids,

### Food insecurity indicators

Data collectors assessed for the presence of a food insecurity during the previous four week period. Out of the 15,159 households surveyed, 8488 (55.99%) were food secure and the rest 6671 (44.01%) were food insecure access. Out of the food insecure households, 1207 (18.09%) were mildly food insecure, 4079 (61.05%) were moderate, and 1385 (20.76) were severely food insecure. In addition, using HHS 14,316 (94.44%) households had little to no hunger, 738 (4.87%) had moderate hunger, and 105 (0.69%) of the households had severe hunger.

Food insecurity had different manifestations in different households. Among all the households, 6296 (41.53%) were forced to limit the variety of food they ate or to have repetitive diet of the same food, and in 514 (3.39%) of the households, some individuals spent the whole day without eating at all (Table 2).

**Table 2:** Frequency of occurrence of behaviors, experiences, and conditions indicating food deficit and hunger in households at DHDSS, 2016

Condition	Frequency of occurrence (percent)			
	Rarely (1–2 times per 4 wk)	Sometimes (3–10 times per 4wk)	Many times (&gt; 10 times per 4 week)	Total households affected
Worried the family wouldn’t get enough food	2078(27.7)	3609(48.1)	1816 (24.2)	7503 (100)
Any of the family members were not getting the food they want	1989(30.36)	3448(52.63)	1114 [17]	6551(100)
Any of the family members forced to eat limited types of food	1577(25.05)	3193(50.71)	1526(24.24)	6296(100)
Any of the family member forced to eat the food they didn’t want	1441(37.4)	1962(51)	448(11.6)	3851(100)
Any of the family members forced to eat small amount of food	1667(31.1)	2835(52.8)	861(16.1)	5363(100)
Any of the family members forced to decrease meal frequency	1816(36.2)	2506(50.0)	689(13.8)	5011(100)
Complete absence of food at home	597(50.94)	434(37.03)	141(12.03)	1172(100)
Any of the family members spend nights feeling hungry	561(57.42)	333(34.08)	83(8.5)	977(100)
Any of the family members spend the whole day without eating food	319(62.06)	147(28.06)	48(9.34)	514(100)

Food insecurity in households occurred with varying degrees of frequency and affected different numbers of household members. Among the 1172 households where there was a complete absence of food, in 141 (12.03%) food was absent frequently (more than ten times per month). Some individuals in these households reported that due to a lack of food they were spending nights feeling hungry, while others were living without any food consumption for entire days. Among the 977 households in which at least one member reported feeling hungry some nights, 83 (8.5%) of the households experienced this situation more than ten times per month) (Table 2).

### Coping strategies of household food insecurity

**Fig. 1:**
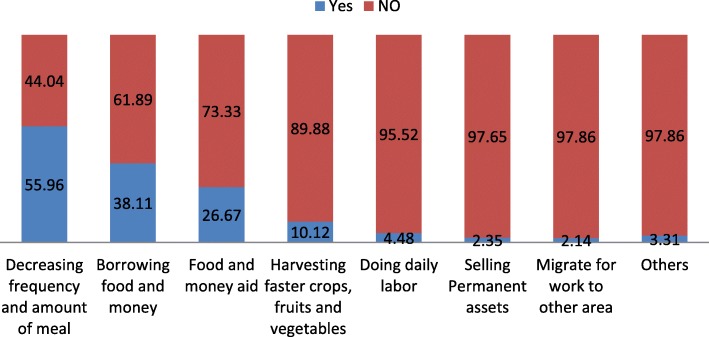
Coping strategies of food insecurity at DHDSS, 2016

Several different coping mechanisms were reported in response to the stress of household food insecurity. In DHDSS, the coping mechanisms identified were: decreasing frequency and portions of meals, borrowing food and money, receiving food and money aid, selling permanent assets, harvesting faster crops, migrating for work, engaging in labor jobs, becoming involved in small businesses, creating hand-made crafts, begging, and selling wood and charcoal. The most common coping mechanism employed by households in the study was to decrease the usual meal frequency and the amount of food. This strategy was used by 3733 (55.96%) households out of the total 6671 households affected by food insecurity **(**Fig. 1**).**

Many households (2542 (38.11%)) borrowed money and/or food to cope with periods of food insecurity. Households reported asking their neighbors, friends and relatives for food and money. In some cases, households described asking local landlords and moneylenders for high-interest, short term loans. Government supported micro-finance schemes were also described.

There was a significant variation across different climatic zones in the coping mechanism they use. Twenty four percent of people who live in low-lands used borrowing food and money as a coping strategy while 14.81% and 12.01% of the mid-altitude *(weyina dega)* and high-landers respectively used this strategy (*P*-value &lt; 0.001). In the contrary, reducing meal frequency and amount was used by 46.05% of low-landers while 67.42% and 60.30% of mid-altitude and high-land dwellers used this strategy respectively (*P*-Value&lt; 0.001). There was no statistically significant difference between other coping strategies across different climatic zones.

In this study, households experiencing food insecurity would also receive food and money aids. Food aid was received in 1779 (26.67%) of the households, with 56 (3.15%) receiving urgent food aid, 1094 (61.5%) receiving safety net aid, 42 (2.36%) receiving aid after disaster, and 544 (30.67%) receiving aid due to aging and illness. The remaining 43 (2.42%) received other aids.

Harvesting faster crops, fruits and vegetables was used by 675 (10.12%) households who had food insecurity. This strategy was mainly used by rural residents. Out of the rural people who had food insecurity, 10.36% used this strategy while only 1.22% of the urban residents used the strategy (*P*-value&lt; 0.001).

Some households used only one strategy for coping with food insecurity while others used a mix of coping mechanisms.

The most popular single coping mechanism, used in1957 (29.34%) households, was decreasing the frequency and amount of meals. This was followed by borrowing food, which was reported by 983 (14.74%) households. The combination of these two mechanisms was used by 680 (10.19%) households.

### Reducing amount and frequency of meal

Households respond to food insecurity by managing the remaining food in their home. This often includes skipping one or more of their usual meals (breakfast, lunch, or dinner). In addition, some households may manage the food they have by eating smaller amounts than they would otherwise eat. As mentioned above, among the 6671 households in DHDSS that experienced insecurity of food, 3733 (55.96%) of them used this strategy as a coping mechanism. This mechanism was used by a majority of the female headed households (62.66%) and urban dwellers (71.13%) (Table 3).

**Table 3:** Socio-demographic determinants of using reducing the amount and frequency of meal as a coping strategy of food insecurity at DHDSS, 2016

Variables	Reduced the amount and frequency of meal		Crude OR(95% CI)	Adjusted OR(95% CI)
	Yes	No		
Household size (Continuous)			0.93 (0.90, 0.95)	^a^
Sex of the household head				
Female	1293(61.66)	804(38.34)	1.41 (1.27, 1.56)	^a^
Male	2440(53.34)	2134 (46.66)	1	
Residence				
Rural	2585(51.12)	2472(48.88)	1	
Urban	1148(71.13)	466(28.87)	2.36 (2.08, 2.66)	2.07 (1.74, 2.46)
Wealth status				
Low income	1440(59.8)	968(40.2)	1.28 (1.13, 1.46)	^a^
Middle income	1429(53.92)	1221(46.08)	1.01 (0.89, 1.14)	
Better income	836(53.69)	721(46.31)	1	
Household Head’s level of education				
Unable to read and write	2249(55)	1837(45)	1	
Able to read and write	807(53.2)	710(46.8)	0.93 (0.82, 1.04)	^a^
Primary education	326(58.63)	230(41.37)	1.16 (0.97, 1.38)	
Secondary education	301(68.72)	137(31.28)	1.79 (1.45, 2.22)	
Tertiary education	50(67.57)	24(32.43)	1.70 (1.04, 2.78)	
Marital status of household heads				
Married	2281(52.58)	2057(47.42)	1	1
Single	230(69.49)	101(30.51)	2.05(1.61, 2.61)	1.21 (0.82, 1.78)
Divorced	522(64.29)	290(35.71)	1.62 (1.39, 1.89)	1.22 (0.92, 1.61)
Widowed	578(56.72)	441(43.28)	1.18 (1.03, 1.36)	1.04 (0.79, 1.37)
Separated	122 (71.35)	49(28.65)	2.25(1.60, 3.14)	1.60(1.07, 2.39)
House hold head’s occupation				
Farmer	2706(52.56)	2442(47.44)	1	
Student	178(69.53)	78(30.47)	2.06(1.57, 2.70)	^a^
All types of paid job	643(69.66)	280(30.34)	2.07(1.78, 2.41)	
Unemployed	81(65.32)	43(34.68)	1.70(1.17, 2.47)	
Others	125(56.82)	95(43.18)	1.18 (0.90, 1.56)	
Climatic Zone				
Hot/Lowland	1120	1312	1	1
Mid-altitude	540	261	2.42 (2.05, 2.87)	2.46 (2.08, 2.92)
Cold/Highland	2073	1365	1.78 (1.60, 1.98)	1.22 (1.08, 1.38)

^a^Not statistically significant

The odds of using reducing amount and frequency of meal as a coping strategy in the face of food insecurity was 2.07 times higher among urban respondents than those who live in rural area (AOR 2.07: 95% CI 1.74, 2.46). Households led by individuals who listed their relationship status as “separated “were 1.60 times more likely to use reducing amount and frequency of meal as a coping strategy than those led by married household heads (AOR 1.60: 95% CI 1.07, 2.39). People who live in mid-altitude areas *(weyina dega)* were 2.46 times more likely to use reducing amount and frequency of meal as a coping strategy than low-land people (AOR 2.46: 95% CI 2.08, 2.92). High-land dwellers are 1.22 times more likely to use reducing amount and frequency of meal as a coping strategy than low-land dwellers (AOR 1.22: 95% CI 1.08, 1.38).

## Discussion

This study aimed to explore food insecurity and coping mechanisms used by survey respondents in the Dabat District. Food insecurity four weeks prior to the data collection was reported by 44.01% of the households, and 36% of the households were suffering from moderate to severe food insecurity. This finding is lower than studies done in Southern Ethiopia, and South Gondar, Ethiopia [27–29]. This could be explained by the seasonal variation in collecting the data. The other studies were done in the pre harvest season when food shortage is relatively higher [30]. Many of the households that had food insecurity reported using single or multiple coping strategies to respond to the problem of lack of food. Coping strategies used included decreasing the frequency and portions of meals, borrowing food and money, receiving food and money aid, selling permanent assets, harvesting faster crops, fruits and vegetables, migrating for work to other areas, doing daily labor, becoming involved in small businesses, doing different hand crafts, begging, and selling wood and charcoal.

Decreasing meal frequency and amount was the most commonly used strategy, which coincides with studies conducted in Nigeria, Uganda and Ghana [14, 16, 17]. In response to the severe insecurity of food in their household, people often may not have other choices, and stay hungry and try to manage the small amount of food in their household. People may use this strategy over borrowing or begging, in light of low productivity and lack of work opportunities. It indicates that, the people are getting undernourished [9, 13] and which exacerbates the burden of malnutrition and related diseases in the area.

People who live in urban areas and people who are separated from their partners were more likely to use this coping strategy. This may be because rural dwellers have farm lands on which to harvest vegetables and other fast growing crops. In this study, harvesting fast growing crops was also a strategy mostly used by rural people. In addition, in the culture of the rural community, it is common practice to help neighbors, friends, and family in time of food insecurity. In urban areas, people are busier and may be less aware of those who need food support.

Heads of household who were living separately used this mechanism more often than married ones. Household heads who report being separated may experience less support, confidence, stability, and efficiency in their work and activities of daily living than those who report being married [31].

Borrowing food and money were also commonly used coping strategies. This is consistent with findings of previous studies conducted in Ethiopia, as well as in Nigeria, and South Africa [5, 16, 17]. When there is insecurity of food at the household level, people often borrow food or money from their neighbors or government This report of borrowing implies the presence of some richer person/lender and micro-financing schemes may also play a role in supporting people who experience food insecurity.

Receiving food and money aid was another common coping strategy. Food aid programs are helping people get relief from hunger [32]. Harvesting fast growing crops such as vegetable and fruit gardens, seeking work as daily labor, and selling permanent assets are coping strategies that have considerably contributed to alleviating food insecurity.

Climatic zone had a significant effect in the coping strategies people use. People who were living in lower altitudes (*Kola)* were more likely to use borrowing as a coping strategy while the mid-altitude *(Weyina-dega)* and high-landers *(dega)* were more likely to use reducing meal amount and frequency as a coping strategy. This might happen due to low-lands are more easily accessible, more cultivatable, and more productive [6]; and people who have food insecurity in low-lands could have better chance to get an assistance from their neighborhoods [33], and to sell the property they have than to reduce the amount and frequency of their meal.

The high burden of food insecurity in these populations needs critical attention to prevent its social, economic, political, and health impacts. With the existing high burden of malnutrition in Ethiopia [34], the high burden of food insecurity and using reducing the amount and frequency of meal as a major coping strategy would bring a big burden on the country. It increases malnutrition and related infectious and non-infectious diseases [35, 36]; decrease productivity [37]; decrease school attendance and educational attainment [38]; and thereby it affects the growth and development of the country [39, 40]. It shows how working on food security is crucial and very important.

Therefore; targeted activities are recommended in order to alleviate the burden of food insecurity in this area. Measures to increase land productivity may be helpful for households in rural areas. In addition to fast growing crops and vegetables, using irrigation is also recommended to increase land productivity. To decrease dependency on crop production, it would be beneficial to invest in other kinds of farming, such as poultry, honey, and diary. Micro finance schemes should be strengthened, to improve access to saving and lending at the household level. Finally, increased access to job opportunities, especially in the urban area where there is little land to harvest, would minimize the negative impacts of food insecurity.

Although this study showed a representative sample of households in the Dabat district, and is assumed to represent the larger area of northwest Ethiopia, there are limitations worth highlighting. We believe that gathering additional data using qualitative methods could strengthen our findings by providing deep exploration into the ways in which households respond and cope with food insecurity.

## Conclusion

Food insecurity in the study area was very high**.** In the response to a food insecurity, households reported using various coping strategies which included decreasing meal frequency and portions (reducing amount and frequency of meal), borrowing food and money, receiving food and money aid, selling permanent assets, harvesting faster crops, migration of the household head in search of work, involvement in daily labor, getting involved in small business activities, making and selling hand crafts, begging, and selling wood and charcoal Decreasing meal frequency and portions or reducing amount and frequency of meal was the most commonly reported coping mechanism and it was significantly associated with residence and marital status. During times of severe insecurity of food in their households, people preferred to stay hungry in order to manage the small amount of food they had. This is an indicator that people may become undernourished, which may exacerbate the burden of malnutrition and other related diseases in the area.
